# Effectiveness of a Cognitive Behavioral Coaching Program Delivered via Video in Real World Settings

**DOI:** 10.1089/tmj.2019.0313

**Published:** 2021-01-08

**Authors:** Anita Lungu, Matthew S. Boone, Shih Yin Chen, Connie E. Chen, Robyn D. Walser

**Affiliations:** ^1^ Lyra Health, Burlingame, California, USA.; ^2^ Department of Psychiatry, University of Arkansas for Medical Sciences, Little Rock, Arkansas, USA.; ^3^ Clinical Science Department, University of California Berkeley, Berkeley, California, USA.; ^4^ Dissemination and Training Division, National Center for PTSD, Palo Alto, California, USA.

**Keywords:** behavioral health, commercial telemedicine, telehealth, e-health

## Abstract

***Background:*** Many employees experience high levels of stress in the workplace, which negatively impact their productivity and well-being. Effective stress management interventions exist, but are inaccessible due to insufficient numbers of mental health providers, long waiting times to initiate care, high out-of-pocket cost of care, and stigma related to receiving psychotherapy.

***Introduction:*** The purpose of this study was to test the efficacy, in real-world circumstances, of a structured, cognitive behavioral coaching (CBC) program delivered through video or telephone.

***Materials and Methods:*** Retrospective data on 289 subjects who had sought support for emotional health through a behavioral health benefit offered through employers were examined. Changes in perceived stress and well-being over the course of the program were measured using the Perceived Stress Scale (PSS) and Warwick–Edinburgh Mental Well-being Scale (WEMWBS), respectively. Rates of reliable change and satisfaction with the coaching program were also assessed.

***Results:*** Scores on both the PSS and WEMWBS improved between baseline and follow-up. Approximately 61.9% (n = 289) of participants demonstrated reliable improvement on either measure.

***Discussion:*** CBC is a promising intervention that has the potential to significantly expand access to effective and more affordable interventions for emotional health care.

***Conclusions:*** Coaching, when delivered by accredited professionals trained in cognitive behavioral theory and interventions and working in real-world settings, can be efficacious in decreasing perceived stress and increasing well-being when delivered through video or telephone.

## Introduction

Employee's health and well-being are important for employees, their families, peers, and the success of their organizations. Modern organizations, under pressure to stay competitive in a global and complex economy, are often characterized by a fast pace of change, persistent uncertainty, and intense workloads, which can place high demands on employees.^1–3^ When employees perceive environmental demands as taxing or exceeding their capacity to adapt and cope effectively, they may experience high levels of stress.^4^

In the U.S. workforce, 65% of employees identify work as a significant source of stress and more than one-third report chronic work stress.^5^ High stress can trigger psychological, behavioral, and biological responses, placing an individual at an increased risk for physical illnesses such as cardiovascular and inflammatory diseases, AIDS/HIV, cancer, and Alzheimer's disease^6–8^ and mental illnesses such as major depression and anxiety.^9,10^ High stress is associated with increased presenteeism (being present at work, but not fully performing duties), absenteeism from the workplace, employee turnover, and disability leave.^11–14^

Fortunately, occupational stress management interventions have been found to be effective, with effect sizes in the moderate to large range, and with cognitive behavioral approaches consistently showing the largest effect.^15^ However, these interventions can be difficult to access for many employees due to scarcity of qualified mental health providers, stigma associated with psychotherapy,^16^ long wait-lists, high out-of-pocket cost of care, and additional transportation time and costs required to attend appointments in person.^17^

### Closing the Gap in Care: Coaching as an Additional Option

Coaching has emerged as a new intervention that applies behavioral science to help clients improve their well-being and performance. It assists clients in reaching desired goals by focusing on growth, values, meaning, self-awareness, and self-actualization. Coaching targets individuals who may be at increased risk for, but do not have, clinically significant mental health problems, and as such, it may circumvent the stigma associated with receiving psychotherapy. Since advanced professional degrees and state licensing are not required for practicing coaching, it has the potential to broaden the number of providers available for working with clients in distress who do not suffer from mental health disorders. In the absence of effective, evidence-based coaching interventions, such clients would likely utilize psychotherapy, impacting the availability that mental health providers could offer to clients who *do* suffer from mental health disorders.

At the same time, coaching poses several challenges. Coaching is less specialized than therapy in addressing different concerns. In recent surveys, for instance, coaches identified more than 38 approaches included in their work (e.g., positive psychology, cognitive behavioral, mindfulness, solution-focused, strength-based, and goal-focused approaches).^18,19^ Studies evaluating coaching oftentimes lack an operational definition of what techniques were included in the interventions.^20,21^ The wide diversity of techniques, less intensive training,^16^ and lower external regulation for the coaching profession compared with psychotherapy can lead to a lot of variation in the quality of services delivered to clients.

### Coaching as an Evidence-Based Approach

Proponents of an evidence-based practice approach for coaching highlight the benefits that coaching offers and encourage coaches to make use of findings from evidence-based psychotherapy^22^ within the coaching context.^23^ Cognitive behavioral coaching (CBC) has emerged as a promising avenue to deliver cognitive behavioral-based interventions in a coaching context. Similar to the rest of the coaching field,^24^ data on the effectiveness of CBC offered at scale in real-world workplaces are nascent and results vary.^25^ Enabling CBC to reach its potential and improve stress management and well-being at scale requires supporting coaches to deliver cognitive behavioral interventions and implementing strong quality assurance protocols, as well as collecting and analyzing data to evaluate program outcomes.

The present study details the implementation, in a real-world setting, of a CBC program targeting stress management and well-being and delivered through video or telephone. Retrospective data from employees and dependents who received these services through Lyra Health are analyzed. Our hypothesis was that clients receiving coaching services would show significant reductions in perceived stress and significant improvements in well-being, as measured by the Perceived Stress Scale (PSS) and Warwick–Edinburgh Mental Well-being Scale (WEMWBS), respectively.

## Materials and Methods

### Overview

This was a retrospective study using existing registry data from Lyra Health and its partners for delivering coaching services. Lyra Health offers a behavioral health benefit to companies through which employees and dependents have access to CBC. Coaches offering the CBC program are trained in accredited International Coaching Federation (ICF) programs and vetted through extensive reviews and interviews for their commitment and ability to provide coaching services aligned with a CBC perspective. Historically, 3% of coaches who apply to Lyra's coaching network have been accepted. Once part of the program, coaches undergo intense training in cognitive behavioral principles.

### Participants

Prospective clients interested in receiving support for stress reduction and/or emotional distress went through an online onboarding process, selecting the symptoms experienced, their impact on their general functioning, and their interest in receiving care through video. A machine learning model applied to participant responses predicted each participant's scores on the GAD-7 (clinical cutoff of 8 and above) and PHQ-9 scales (clinical cutoff of 10 and above).^26–29^ The machine learning model was developed based on users completing the triage flow and then completing PHQ-9 and GAD-7 questionnaires. Consequently, the triage flow responses constitute the model features and the questionnaire severity ranges are the variables that the model is trained to predict. On the gold standard set, the model's precision and recall for the subclinical category are ∼80%.^30^ Participants were not administered the PHQ-9 and GAD-7 scales directly. If subjects were interested in video care and the predicted scores were lower than the clinical cutoffs, they were offered the coaching program as a care option. Alternatively, if clients' predicted scores were above the cutoff, they were not offered the coaching program and instead were offered therapy or access to a self-care online program. Before the first coaching session, clients completed the K-6 questionnaire^31^ as an additional screening assessing their appropriateness to receive coaching. Clients scoring above 12 on the K-6 or individuals manifesting symptoms of high distress during the first or subsequent coaching sessions were referred out of the coaching program to therapy with a licensed therapist (see *Table 1* for CBC exclusion criteria).

**Table 1.** Exclusion Criteria from the Cognitive Behavioral Coaching Program

<18 Years of age
K6 score ≥12
PHQ-9 score as predicted by machine learning algorithm ≥10
GAD-7 score as predicted by machine learning algorithm ≥8
Already receiving psychotherapy
Seeking care for substance use, gender identity issues, parenting skills, traumatic stress, major mental illness, and physical or sexual abuse
Thoughts of suicide, homicide, or self-harm, current or in the last 6 months
Reported use of medication for behavioral health concerns

**Fig. 1.**
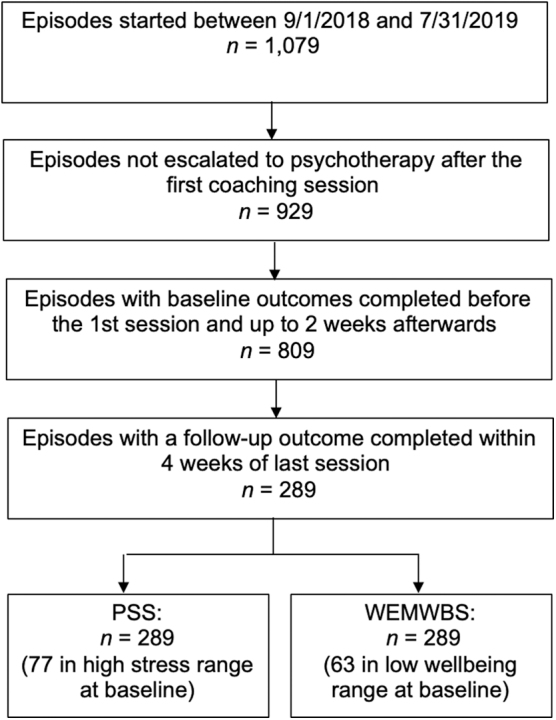
Participant flow.

Participants, who started the coaching program between September 1, 2018, and July 31, 2019, were included in the study. Participants were either employees or dependents from companies that had partnered with Lyra Health. Participants were included in the data analysis if they received at least one coaching session and completed the PSS and WEMWBS outcome measures at baseline, within 4 weeks before or 2 weeks after their first coaching session, and at postintervention within 4 weeks after their last coaching session. Of 1,079 participants who started CBC, 150 (13.9%) were escalated to psychotherapy. Of the remaining 929 participants, 809 (87%) completed baseline assessments and 289 (31%) also completed postintervention outcomes, as defined above (*Fig. 1*).

The outcome measures were completed on a mobile phone app as electronically secure questionnaires. This study was classified as exempt from human subjects review by the Western Institutional Review Board (WIRB).^32^

### Coaching Training Program

Once accepted, coaches undergo intensive training in CBC, designed and delivered over several months by licensed mental health professionals who are experts in cognitive behavior therapy (CBT). The training comprises a lecture and discussion; small group and one-on-one skill practice; videos outlining and demonstrating CBT skills; written feedback on audio recordings of sessions; personal practice of CBT skills; creating and receiving feedback on case presentations; individual consultation; and ongoing group consultation. Consultations were conducted through videoconference and facilitated by licensed mental health professionals and/or senior coaches with extensive practice with the model.

### Coaching Program

Participants met with coaches for up to six 45-min sessions through video or telephone, with the option of adding a booster session at the end. Coaches augmented their work with digital materials (informational handouts, worksheets, blogs, and videos) that were sent to the client following sessions. Clients could message their coaches through an Health Insurance Portability and Accountability Act-compliant mobile app to ask questions about their assigned homework. The core skills were drawn from intervention models in the CBT tradition, including traditional CBT,^33^ acceptance and commitment therapy,^34^ and dialectical behavior therapy^35^ (*Table 2*). Coaches were encouraged to adhere to the coaching competencies outlined by the International Coaching Federation (ICF), such as establishing trust and intimacy, powerful questioning, planning, and goal setting.^36^

**Table 2.** Examples of Skills Included in the Cognitive Behavioral Coaching Program

SKILLS	DEFINITION
Values	Identifying qualities of ongoing action that bring meaning and vitality to one's life.
Mindful awareness	Turning attention to the present in a deliberate nonjudgmental way to observe the ongoing stream of thoughts and feelings as they arise.
Cognitive reappraisal	Identifying ineffective patterns of thinking (i.e., cognitive distortions such as catastrophizing and “should statements”), examining the evidence to support them, and generating more fact-based responses.
Cognitive defusion	Noticing thinking as it occurs and undermining the impact of thoughts to organize behavior when doing so is not useful.
Acceptance	Adopting a willing and receptive stance toward thoughts and feelings, as well as the circumstances that give rise to them. Foregoing unnecessary or ineffective attempts at suppression and control of internal experience.
Opposite action	Acting in opposition to an urge associated with an emotion (e.g., the desire to withdraw associated with sadness) to reduce the intensity of the emotion and/or choose behavior that is more effective for one's goals.
Distress tolerance	Strategically distracting oneself from intense emotions when the situations that evoke them are not immediately changeable.
Effective communication	Expressing oneself in ways that are effective for one's goals in relationships.

### Measures

Used to guide clinical appropriateness for CBC, the K6^31^ is a six-item dimensional measure of nonspecific psychological distress on a five-point Likert scale, with scores ranging from 0 to 24. Clients scoring above 12, indicating more severe distress, were transferred to psychotherapy.

Outcome assessments consisted of the PSS and WEMWBS, well-validated measures of perceived stress and well-being.^37,38^ The PSS includes 10 items rated on a 5-point Likert scale, with scores ranging from 0 to 40; higher scores represent increased perceived levels of stress. The WEMWBS includes 14 items rated on a 5-point scale, with scores ranging from 14 to 70; higher scores represent increased well-being. For a subset of study analyses, a cutoff of 22 was used for the PSS to indicate the presence of high perceived stress (value calculated in prior research as a standard deviation above the mean of a large working population^39^). Similarly a value of 40 and below was used for the WEMWBS to indicate the presence of low well-being.^38,40^

At the end of each session, participants answered a single question to measure satisfaction (“How satisfied are you with your relationship with your coach?”) using a 1- to 5-star rating system. Participants were informed that their answers would not be shared with the coach directly. At the end, participants rated their satisfaction with the entire program (“How satisfied are you with the coaching program?”) on a four-point scale (from “Very Dissatisfied” to “Very satisfied”) and whether they would recommend the program to others (“How likely are you to recommend your Lyra coach to someone with similar problems?”) on a four-point scale (from “Not at all likely” to “Extremely likely”).

### Analyses

Paired *t*-tests were used to test differences between baseline and postscores on the PSS and WEMWBS. Cohen's *d* was calculated as a measure of effect size.^41^ The analyses were conducted in R.^42^ The number of participants who demonstrated reliable change (RC) on either measure was calculated using the RC index proposed by Jacobson and Truax,^43^ with values for PSS and WEMWBS calculated in prior research.^39,44^ The RC index indicates whether a subject made substantial improvement in symptomatology, change that is beyond what could be attributed to chance (measurement error). To better assess the impact of care on subjects presenting with elevated distress at baseline, we also performed *t*-test analyses and calculated the RC only for individuals with PSS scores greater or equal to 22 or WEMWBS scores lower or equal to 40.

## Results

Of the 289 participants included in analyses, 54.3% (*n* = 157) identified as female and 45.3% (*n* = 131) identified as male. The mean age of participants was 33.9 years (standard deviation [*SD*]* =* 8.1). On average, the baseline score on the K6 was 6.37 (*SD* = 3.15).

The 289 participants were seen by 34 coaches. On average, each therapist saw 8.62 (*SD =* 10.06) participants. The average number of CBC sessions delivered across the course of care was 5.79 (*SD* = 1.06). The average number of weeks that clients spent in care was 9.31 (*SD* = 4.54).

### Perceived Stress Scale

Results of the paired *t*-test revealed that the score on the PSS decreased significantly between baseline (*M* = 18.15, *SD* = 5.22) and follow-up (*M* = 13.63, *SD* = 4.91), with participants improving an average of 4.52 points on the PSS (*SD* = 5.59), *t*(288) = 13.76, *p* < 0.001. Cohen's *d* suggested a large treatment effect size on the PSS, with *d* = 0.89 (*Table 3*). Improvement in stress scores was observed in 221 participants (76.47%), and 119 participants (41.18%) showed reliable improvement. Deterioration was observed in 52 participants (17.99%), and 10 participants showed reliable deterioration (*Table 4*).

**Table 3.** Baseline and Postcompletion Perceived Stress Scale and Warwick–Edinburgh Mental Well-being Scale Scores

					95% CONFIDENCE INTERVAL OF THE DIFFERENCE				
MEASURE	N	BASELINE OUTCOME, MEAN (SD)	FOLLOW-UP OUTCOME, MEAN (SD)	PAIRED DIFFERENCES, MEAN (SD)	LOWER	UPPER	T-VALUE (df)	p-VALUE	COHEN'S d
PSS									
All	289	18.15 (5.22)	13.63 (4.91)	4.52 (5.59)	3.88	5.17	13.76 (288)	<0.001	0.89
PSS ≥22	77	24.19 (2.16)	16.36 (4.80)	7.83 (5.16)	6.66	9.00	13.31 (76)	<0.001	2.09
WEMWBS									
All	289	47.23 (8.05)	55.69 (7.42)	8.46 (8.37)	7.49	9.43	17.17 (288)	<0.001	1.09
WEMWBS ≤40	63	36.98 (2.90)	51.49 (8.30)	14.51 (8.09)	12.47	16.55	14.23 (62)	<0.001	2.20

PSS, Perceived Stress Scale; WEMWBS, Warwick–Edinburgh Mental Well-being Scale.

**Table 4.** Rates of Improvement and Deterioration on Perceived Stress Scale and Warwick–Edinburgh Mental Well-being Scale

	PSS (N = 289)	PSS ≥22 (N = 77)	WEMWBS (N = 289)	WEMWBS ≤40 (N = 63)
Any improvement, *n* (%)	221 (76.47)	73 (94.81)	241 (83.39)	61 (96.83)
Reliable improvement, *n* (%)	119 (41.18)	48 (62.34)	160 (55.36)	52 (82.54)
Any deterioration, *n* (%)	52 (17.99)	3 (3.90)	41 (14.19)	1 (1.59)
Reliable deterioration, *n* (%)	10 (3.46)	0 (0.00)	6 (2.08)	0 (0.00)

Of the 289 participants, 77 (26.64%) scored in the high stress range on the PSS at baseline (PSS ≥22). Among participants who started in the high stress range, PSS scores decreased significantly between baseline (*M* = 24.19, *SD* = 2.16) and follow-up (*M* = 16.36, *SD* = 4.80), with an average improvement of 7.83 points (*SD* = 5.16), *t*(76) = 13.31, *p* < .001. Cohen's *d* suggested a large treatment effect size on the stress scale, with *d* = 2.09 (*Table 3*). Of the 77 participants with high stress scores, an improvement was observed in 73 participants (94.81%), and 48 participants (62.34%) demonstrated reliable improvement. Deterioration was observed in three participants (3.9%), no participant showed reliable deterioration (*Table 4*).

### Warwick–Edinburgh Mental Well-Being Scale

Of the 289 participants included in analyses, results of the paired *t*-test revealed that the mental well-being scale increased significantly between baseline (*M* = 47.23, *SD* = 8.05) and follow-up (*M* = 55.69, *SD* = 7.42), with participants improving an average of 8.46 points on the WEMWBS (*SD* = 8.37), *t*(288) = 17.17, *p* < 0.001. Cohen's *d* suggested a large treatment effect size on mental well-being with *d* = 1.09 (*Table 3*). Of the 289 participants, 241 participants (83.39%) showed improvements, and 160 participants (55.36%) demonstrated reliable improvement. Deterioration was observed in 41 participants (14.19%), and 6 participants (2.08%) demonstrated reliable deterioration (*Table 4*).

At baseline, 63 participants (21.80%) scored in the low mental well-being range on the WEMWBS. For the 63 participants, the mental well-being scales increased an average of 14.51 points (*SD* = 8.09) between baseline (*M* = 36.98, *SD* = 2.90) and follow-up (*M* = 51.49, *SD* = 8.30), and results of the paired *t*-test revealed that this difference was statistically significant, with *t*(62) = 14.23, *p* < 0.001. Cohen's *d* suggested a large treatment effect size on mental well-being, with *d* = 2.20) (*Table 3*). Of the 63 participants with low mental well-being scores on the WEMWBS at baseline, improvement was observed in 61 participants (96.83%), and 52 participants (82.54%) demonstrated reliable improvement. Deterioration was observed in one participant (1.59%), and no participant showed reliable deterioration (*Table 4*).

### Satisfaction

There were 1,672 coaching sessions offered to 289 participants, of which 49.28% (*n* = 824) of the sessions received satisfaction ratings. Of all participants, 85.12% (*n* = 246) submitted at least one session rating. The mean satisfaction score was 4.91 (*SD* = 0.32, *n* = 824) on the session level. Every participant rated their satisfaction level and how likely they would recommend their coach to someone with similar problems at the end of the episode. Of the 289 participants, 95.16% (*n* = 275) gave a rating of “Very Satisfied” or “Satisfied” in the final assessments and 94.81% (*n* = 274) said they were “Extremely likely” or “Likely” to recommend their coach (*Table 5*).

**Table 5.** Rates of Satisfaction and Recommendation at the End of the Episode

MEASURE	ALL PARTICIPANTS (N = 289)	PSS ≥22 (N = 77)	WEMWBS ≤40 (N = 63)
Satisfaction			
Very satisfied	214 (74.05)	59 (76.62)	41 (65.08)
Satisfied	61 (21.11)	13 (16.88)	18 (28.57)
Dissatisfied	2 (0.69)	0 (0.00)	0 (0.00)
Very dissatisfied	12 (4.15)	5 (6.49)	4 (6.35)
Recommendation			
Extremely likely	220 (76.12)	60 (77.92)	43 (68.25)
Likely	54 (18.69)	14 (18.18)	14 (22.22)
Somewhat likely	12 (4.15)	2 (2.60)	4 (6.35)
Not at all likely	3 (1.04)	1 (1.30)	2 (3.17)

## Discussion

Coaching is a promising intervention when using behavioral science to assist clients in reducing stress and its related mental health issues. In this retrospective analysis, clients receiving coaching care for stress through a six-session CBC program showed significant improvement, with large treatment effects, in stress reduction and well-being, with more than 60% of participants demonstrating RC. These outcome data set the stage for growth in the capacity to provide an intervention for stress-related difficulties at work and a stepped care approach.

CBC holds the potential to meaningfully expand access to quality stress-related emotional health care, reducing the barriers to helpful interventions. The current study also adds to the literature supporting coaching as a useful tool for improving well-being in individuals seeking care, who do not suffer from mental health disorders, which is more cost-effective than psychotherapy and more easily disseminated. For instance, since coaches do not require state-specific licensure to practice, CBC has the potential to unlock a large pool of professionals who can operate flexibly across state lines and treat clients in difficult-to-reach areas. Individuals in distress, but scoring in the subclinical range on symptoms of depression and anxiety, were successfully identified from individuals seeking care, referred to the coaching program, and found the coaching program satisfactory. This kind of access may prove useful to organizations who are seeking to reduce presenteeism, absenteeism, and employee turnover, as well as to help individuals adapt, cope, and make a change in ways that best support well-being in demanding environments. Additionally, providing clients who experience significant, but not yet clinical, levels of distress with an effective intervention may prevent them from escalating into the clinical range of symptoms that has increased negative impact on their functioning and may require more lengthy and costly care. It has been argued that even with optimal care and service delivery, less than 30% of the burden of disease attributable to mental disorders could be averted.^45^ As such, a focus on increased utilization of prevention interventions for mental health, in addition to increasing access to treatment for mental health disorders, can bring substantial benefits to individuals in distress, their families, employers, and society overall. Finally, these data may assist those considering how to improve access by decreasing skepticism about coaching due to sparse data on its effectiveness.

Some limitations of the current study should be noted. First, there was no measure of intervention fidelity, so we cannot be certain whether the coaching delivered had high fidelity to CBC principles and techniques in all cases. The coaches in the CBC program received substantial CBT training and access to ongoing consultation, so the results demonstrated here may not generalize to other CBC programs where less clinical training and support are available. The study did not utilize a randomized controlled trial (RCT) design, so we cannot be sure that the results obtained were due to the intervention, as opposed to the passage of time or other factors in the study population. Only 289 (31%) of the original number of participants with episodes in the considered time window, who completed the pre- and postassessments, were included in the analyses. Of the initial group, 150 (13.9%) were escalated to psychotherapy, 120 (11.1%) did not complete a baseline assessment before the first session and up to 2 weeks afterward, and 520 (48.2%) did not have a postcare assessment completed within 4 weeks of the last session. It is possible that participants who did not provide either baseline or postcare assessments were qualitatively different from participants who provided such data. Interpretation of our results should take into account the significant attrition from assessments. However, the effect sizes on both the measure of stress and well-being were large and in the range of those obtained in RCT studies,^15,41^ suggesting that the CBC program provided an effective intervention for clients. The effect sizes obtained were also larger for individuals with more pronounced symptoms, presenting at the high end of the stress scale and low end of the well-being scale, indicating that those clients would see most change as a result of the program.

Despite these limitations, this study contributes to our knowledge of the effectiveness of CBC under real-world conditions. The results suggest that CBC, when coupled with supervision, training, and quality assurance, can be efficacious in decreasing perceived stress and improving well-being. Future research should seek to better understand whether CBC is effective for psychological outcomes other than stress and well-being and whether it reduces other negative by-products of high stress such as presenteeism, absenteeism, or turnover. Exploring the cost-effectiveness of the CBC program compared with traditional psychotherapy is also an area of interest for future research. Future research could also explore the efficacy and effectiveness of CBC with participants starting the program at higher levels of distress. Additional training, supervision, and quality assurance elements (e.g., for identifying and managing clinical risk and lack of clinical progress) would likely need to be in place to make such a program safe and effective for these clients. Additional research is also needed to better understand the mechanisms of change in CBC as well as the contribution of the training and quality assurance components of the program.

## References

[1] Bowling NA, Alarcon GM, Bragg CB, Hartman MJ A meta-analytic examination of the potential correlates and consequences of workload. Work Stress 2015**;** 29:95–113

[2] Dahl MS Organizational change and employee stress. Manag Sci 2010**;** 57:240–256

[3] Schmidt S, Roesler U, Kusserow T, Rau R Uncertainty in the workplace: Examining role ambiguity and role conflict, and their link to depression—A meta-analysis. Eur J Work Organ Psychol 2014**;** 23:91–106

[4] Cohen S, Gianaros PJ, Manuck SB A stage model of stress and disease. Perspect Psychol Sci J Assoc Psychol Sci 2016**;** 11:456–463 10.1177/1745691616646305 PMC5647867 27474134

[5] APA. APA survey finds US employers unresponsive to employee needs. Available at https://www.apa.org/news/press/releases/2013/03/employee-needs Published 2013. (last accessed 1031, 2019)

[6] Cohen S, Janicki-Deverts D, Doyle WJ, et al. Chronic stress, glucocorticoid receptor resistance, inflammation, and disease risk. Proc Natl Acad Sci U S A 2012**;** 109:5995–5999 22474371 10.1073/pnas.1118355109 PMC3341031

[7] Cohen S, Janicki-Deverts D, Miller GE Psychological stress and disease. JAMA 2007**;** 298:1685–1687 17925521 10.1001/jama.298.14.1685

[8] Machado A, Herrera AJ, de PRM, et al. Chronic stress as a risk factor for Alzheimer's disease. Rev Neurosci 2014**;** 25:785–804 25178904 10.1515/revneuro-2014-0035

[9] Siegrist J Chronic psychosocial stress at work and risk of depression: Evidence from prospective studies. Eur Arch Psychiatry Clin Neurosci 2008**;** 258:115 18985307 10.1007/s00406-008-5024-0

[10] Melchior M, Caspi A, Milne BJ, Danese A, Poulton R, Moffitt TE Work stress precipitates depression and anxiety in young, working women and men. Psychol Med 2007**;** 37:1119–1129 17407618 10.1017/S0033291707000414 PMC2062493

[11] Paillé P Perceived stressful work, citizenship behaviour and intention to leave the organization in a high turnover environment: Examining the mediating role of job satisfaction. J Manag Res 2011**;** 3:E1

[12] Gosselin E, Lemyre L, Corneil W Presenteeism and absenteeism: Differentiated understanding of related phenomena. J Occup Health Psychol 2013**;** 18:75–86 23276197 10.1037/a0030932

[13] Jourdain G, Vézina M How psychological stress in the workplace influences presenteeism propensity: A test of the Demand–Control–Support model. Eur J Work Organ Psychol 2014**;** 23:483–496

[14] Dahl J, Wilson KG, Nilsson A Acceptance and commitment therapy and the treatment of persons at risk for long-term disability resulting from stress and pain symptoms: A preliminary randomized trial. Behav Ther 2004**;** 35:785–801

[15] Richardson KM, Rothstein HR Effects of occupational stress management intervention programs: A meta-analysis. J Occup Health Psychol 2008**;** 13:69–93 18211170 10.1037/1076-8998.13.1.69

[16] Bishop TF, Seirup JK, Pincus HA, Ross JS Population of US practicing psychiatrists declined, 2003-13, which may help explain poor access to mental health care. Health Aff Proj Hope 2016**;** 35:1271–1277 10.1377/hlthaff.2015.1643 27385244

[17] Weil TP Insufficient dollars and qualified personnel to meet United States mental health needs. J Nerv Ment Dis 2015**;** 203:233 25816044 10.1097/NMD.0000000000000271

[18] Palmer S, Whybrow A What do coaching psychologists and coaches really do? Results from two international surveys. Invited paper at the 7th International Congress of Coaching Psychology. London, **2017**

[19] Palmer S, Whybrow A Handbook of coaching psychology: A guide for practitioners. London, UK: Routledge, **2018**

[20] Ammentorp J, Uhrenfeldt L, Angel F, Ehrensvärd M, Carlsen EB, Kofoed P-E Can life coaching improve health outcomes?—A systematic review of intervention studies. BMC Health Serv Res 2013**;** 13:428 24148189 10.1186/1472-6963-13-428 PMC4015179

[21] Newnham-Kanas C, Gorczynski P, Morrow D, Irvin J Annotated bibliography of life coaching and health research. Int J Evid Based Coach Mentor 2009**;** 7:39–103

[22] Goodheart C, Kazdin A, Sternberg R, eds. Evidence-based psychotherapy: Where practice and research meet. Washington, DC: American Psychological Association, **2006**

[23] Stober D, Wildflower L, Drake D Evidence-based practice: A potential approach for effective coaching. Int J Evid Based Coach Mentor 2006**;** 4

[24] Theeboom T, Beersma B, Vianen AEM van Does coaching work? A meta-analysis on the effects of coaching on individual level outcomes in an organizational context. J Posit Psychol 2014**;** 9:1–18

[25] Gyllensten K, Palmer S Can coaching reduce workplace stress? A quasi-experimental study. Int J Evid Based Coach Mentor 2005**;** 3:75–85

[26] Kroenke K, Spitzer RL, Williams JB The PHQ-9: Validity of a brief depression severity measure. J Gen Intern Med 2001**;** 16:606–613 11556941 10.1046/j.1525-1497.2001.016009606.x PMC1495268

[27] Spitzer RL, Kroenke K, Williams JBW, Löwe B A brief measure for assessing generalized anxiety disorder: The GAD-7. Arch Intern Med 2006**;** 166:1092–1097 16717171 10.1001/archinte.166.10.1092

[28] Plummer F, Manea L, Trepel D, McMillan D Screening for anxiety disorders with the GAD-7 and GAD-2: A systematic review and diagnostic metaanalysis. Gen Hosp Psychiatry 2016**;** 39:24–31 26719105 10.1016/j.genhosppsych.2015.11.005

[29] Kroenke K, Spitzer RL, Williams JBW, Monahan PO, Löwe B Anxiety disorders in primary care: Prevalence, impairment, comorbidity, and detection. Ann Intern Med 2007**;** 146:317–325 17339617 10.7326/0003-4819-146-5-200703060-00004

[30] Hastie T, Tibshirani R, Friedman J The elements of statistical learning: Data mining, inference, and prediction, 2nd ed. Springer Science & Business Media, **2009**

[31] Kessler RC, Andrews G, Colpe LJ, et al. Short screening scales to monitor population prevalences and trends in non-specific psychological distress. Psychol Med 2002**;** 32:959–976 12214795 10.1017/s0033291702006074

[32] WIRB Home. Available at www.wirb.com/Pages/default.aspx (last accessed 1218, 2019)

[33] Beck AT, Rush JA, Shaw BE, Emery G Cognitive Therapy of Depression. New York, USA: Guilford Press, **1979**

[34] Hayes SC, Strosahl KD, Wilson KG Acceptance and Commitment Therapy, The Process and Practice of Mindful Change. 2nd ed. New York, USA: Guilford Press, 2011

[35] Linehan MM DBT Skills Training Manual, 2nd ed. New York, USA: Guilford Press, 2014

[36] The Gold Standard in Coaching | ICF—Core competencies. International Coach Federation. https://coachfederation.org/core-competencies (last accessed 1217, 2019)

[37] Cohen S, Kamarck T, Mermelstein R A global measure of perceived stress. J Health Soc Behav 1983**;** 24:385–396 6668417

[38] Tennant R, Hiller L, Fishwick R, et al. The Warwick-Edinburgh Mental Well-being Scale (WEMWBS): Development and UK validation. Health Qual Life Outcomes 2007**;** 5:63 18042300 10.1186/1477-7525-5-63 PMC2222612

[39] Ebert DD, Lehr D, Heber E, Riper H, Cuijpers P, Berking M Internet- and mobile-based stress management for employees with adherence-focused guidance: Efficacy and mechanism of change. Scand J Work Environ Health 2016**;** 42:382–394 27249161 10.5271/sjweh.3573

[40] Bianco D Performance of the Warwick-Edinburgh Mental Wellbeing Scale (WEMWBS) as a screening tool for depression in UK and Italy, **2017**

[41] Lakens D Calculating and reporting effect sizes to facilitate cumulative science: A practical primer for t-tests and ANOVAs. Front Psychol 2013**;** [Epub ahead of print]; DOI: 10.3389/fpsyg.2013.00863 PMC3840331 24324449

[42] R: a language and environment for statistical computing. Available at https://www.gbif.org/tool/81287/r-a-language-and-environment-for-statistical-computing (last accessed 1217, 2019)

[43] Jacobson NS, Truax P Clinical significance: A statistical approach to defining meaningful change in psychotherapy research. J Consult Clin Psychol 1991**;** 59:12–19 2002127 10.1037//0022-006x.59.1.12

[44] Maheswaran H, Weich S, Powell J, Stewart-Brown S Evaluating the responsiveness of the Warwick Edinburgh Mental Well-Being Scale (WEMWBS): Group and individual level analysis. Health Qual Life Outcomes 2012**;** 10:156 23270465 10.1186/1477-7525-10-156 PMC3560098

[45] Jacka FN, Reavley NJ Prevention of mental disorders: Evidence, challenges and opportunities. BMC Med 2014**;** 12:75 24886356 10.1186/1741-7015-12-75 PMC4014629

